# Universal strategy for preimplantation genetic testing for cystic fibrosis based on next generation sequencing

**DOI:** 10.1007/s10815-019-01635-2

**Published:** 2019-12-11

**Authors:** Sandrine Chamayou, Maria Sicali, Debora Lombardo, Carmelita Alecci, Carmen Ragolia, Elena Maglia, Annalisa Liprino, Clementina Cardea, Giorgia Storaci, Simona Romano, Antonino Guglielmino

**Affiliations:** Unità di Medicina della Riproduzione - Centro HERA, via Barriera del Bosco n. 51/53, Sant’Agata Li Battiati, 95030 Catania, Italy

**Keywords:** CFTR, Cystic fibrosis, Next generation sequencing, PGT, Preimplantation genetic diagnosis

## Abstract

**Purpose:**

We developed and applied a universal strategy for preimplantation genetic testing for all cystic fibrosis gene mutations (PGT-CF) based on next-generation sequencing (NGS).

**Methods:**

A molecular protocol was designed to diagnose all CF mutations at preimplantation stage. The detection of CF mutations was performed by direct gene sequencing and linkage strategy testing 38 specific SNPs located upstream and inside the gene for PGT-CF. Seventeen couples at risk of CF transmission decided to undergo PGT-CF. Trophectoderm cell biopsies were performed on day 5–6 blastocysts. PGT for aneuploidy (PGT-A) was performed from the same samples. Tested embryos were transferred on further natural cycles.

**Results:**

PGT was performed on 109 embryos. Fifteen CF mutations were tested. PGT-CF and PGT-A were conclusive for respectively 92.7% and 95.3% of the samples. A mean of 24.1 SNPs was informative per couple. After a single embryo transfer on natural cycle, 81.3% of the transferred tested embryos were implanted.

**Conclusions:**

The present protocol based on the entire CFTR gene together with informative SNPs outside and inside the gene can be applied to diagnose all CF mutations at preimplantation stage.

**Electronic supplementary material:**

The online version of this article (10.1007/s10815-019-01635-2) contains supplementary material, which is available to authorized users.

## Introduction

Cystic fibrosis (CF) is the most widespread autosomal recessive disease in the Caucasian population as one Caucasian person in 25 is a carrier [1], and the incidence is 1 in 3500 live births [2]. The gene responsible for CF is named the cystic fibrosis transmembrane conductance regulator (CFTR) and was sequenced in 1989 [3, 4]. It is located in position 7q31.2 and is compound of 27 exons. The CFTR gene encodes for cyclic adenosine monophosphate-dependent chloride channel located in the apical membrane of secretary epithelial cells [5, 6]. Inactivity of the transmembrane protein due to mutation has a consequence hyper-viscosity of epithelial secretions. CF is mainly due to point mutations or few bases deletion of the CFTR gene. A total of 2067 genomic variants were recorded in the first Cystic Fibrosis Mutation Database [7]. On the 11 March of 2019, the causing mutations have been recalculated as 383 [8]. This wide genetic variability and the combination with downstream polymorphisms make the clinical symptomatology of the disease not always predictable and varying from a mild clinical expressivity with atypical pancreatitis and bronchiectasis to severe health consequences including chronic pulmonary obstruction, infections, exocrine pancreatic insufficiency, elevated sweat electrolytes, and death [9].

CF is also responsible for the alteration of the genital tract [10–13]. In males, the absence or severely reduced activity of CFTR protein due to genetic mutations can lead to excessive viscosity of the epididymal fluid and results in male infertility [11, 13] with congenital bilateral absence of vas deferens (CBAVD) [14, 15] when associated with (TG)m and Tn polymorphisms loci in intron 8 at the splice acceptor site of exon 9 of CFTR gene [16, 17]. In 2009, the latest practice European guidelines for molecular genetic of CF and CFTR-related disorders were published [18].

CF was the first autosomal monogenic disease to be diagnosed at preimplantation stage [19]. Several preimplantation genetic diagnosis (PGD) centers reported their long-term experience [20, 21] on a limited number of CF mutations. From the first report of ESHRE PGD Consortium [22] to the last detailed data collection [23], CF is among the first indications for PGD, today renamed as “preimplantation genetic testing” (PGT).

According to the latest guidelines for PGT for CF (PGT-CF), the molecular strategy should be based on PCR amplification and diagnosing the causing mutation(s) together with linked polymorphisms within or close to the CFTR gene [24]. Recent technological advances such as next generation sequencing (NGS) enlarged the spectrum of detectable mutations [25, 26], and PGT can now be accessible for all known CF mutations.

The aim of our study was to develop universal strategy and protocol for PGT for all known CF mutations sequencing the entire CFTR gene by NGS with informative single nucleotide polymorphims (SNPs) in upstream and inside the gene. PGT-CF together with preimplantation genetic testing for aneuploidy (PGT-A) was applied on couples from the eastern Sicily and at risk of CF transmission.

## Materials and methods

Each step of the protocol and the sequence of each step have been approved by the Institutional Review Board. All participants have been informed and gave written consent for CF screening and all aspects of PGT-CF and PGT-A.

### Couples undergoing PGT-CF

From July 2014 to December 2018, 1052 infertile couples living in eastern Sicily were screened for CFTR mutations from blood samples. Ten of them resulted couples of both carriers of CFTR mutation(s). These 10 couples plus 7 additional fertile Sicilian couples that previously knew to be at risk of CF transmission requested to undergo PGT-CF with PGT-A.

### CF screening from blood

The present protocol has been previously validated in an international network for CFTR gene mutation detection using NGS [25].

Genomic DNA extraction from 200 μl of peripheral blood was performed using the standard protocol of the High Pure PCR Template Preparation Kit (Roche Diagnostics). The DNA samples were then quantified using the Qubit® 2.0 Fluorometer (Thermo Fisher Scientific), in order to proceed with library preparation.

A total of 5 ng of DNA extracted from each sample was used to prepare amplicon libraries through a multiplex PCR with specific primers for the CFTR gene, according to the Ion Ampliseq CFTR Panel. The libraries were prepared with the AmpliSeq Library Kit 2.0 (Life Technologies-Thermo Fisher, Carlsbad, USA) and barcoded with IonXpress Barcode Adapter Kit 1–16 or 17–32 (Life Technologies). After quantification, each library was normalized to 100 pM. All libraries were mixed to obtain a final concentration of 8 pM and clonally amplified with the Ion PGM™ Hi-Q™ View OT2 Kit on the Ion OneTouch 2 System. Up to 16 enriched libraries were loaded on Chip 16 V2. DNA sequencing was performed with the Ion PGM™ Hi-Q™ View Sequencing Kit on the Ion Personal Genome Machine. The updated Torrent Suite Software was used for base calling and mapping on human genome reference sequence hg19.

After sequencing, unaligned tab-delimited text files (.BAM) containing sequence alignment data were uploaded to the cloud-based Ion Reporter Software v.5.10.2.0 (Life Technologies), and the alignment of the sequences was visualized with the Integrative Genomic Viewer (IGV, Broad Institute) using the human genome hg19 as a reference. The variant analysis was processed using the dedicated workflow *AmpliSeq CFTR single sample* for the identification, filtering, and annotation of the CFTR variants affecting each sample.

### Validation and set-up for PGT-CF

Before clinical application, the present protocol of CF mutation and SNP detections (and linkage for families) was validated on genomic DNA obtained from 64 known carriers, two family’s trios (parents and child), and 15 carrier couples with relatives (parents and siblings of each member of the couple) for 24 mutations. The protocol of CFTR gene and SNP amplification and detection was also performed and validated on single cells obtained from the IVF laboratory (oocyte and embryonic cells from arrested embryos).

Once a couple at risk for CF transmission decided to undergo PGT-CF, set-up for PGT-CF was performed from DNA extracted from peripheral blood of direct relatives (parents, children, or siblings) of each member of the couple in order to determine all the informative single nucleotide polymorphisms (SNP) close or inside the CFTR gene, and to verify the CFTR mutations when previously determined in another genetic laboratory. It was also possible to follow allele segregation by analyzing 2-day arrested embryos from the same PGT-CF cycle.

Thirty-eight SNPs have been selected with a high degree of heterozygosis, minor allele frequency (MAF) values higher than 0.238, so with a high likelihood of informativity for genetic linkage analysis. For the SNP design, the dbSNP 138 (http://bioq.saclab.net/query/submit.php?db=bioq_dbsnp_human_138) was used in combination with the UCSC Genome Browser (https://genome.ucsc.edu). The design of the amplicons with their respective primers was realized using the Ion AmpliSeq Designer Software (https://www.ampliseq.com-Life Technologies). Given the extension of the CFTR gene (188.702 bp–NCBI Gene Database https://www.ncbi.nlm.nih.gov/gene), 34 SNPs evenly distributed within the gene and 4 SNPs at a gene distance of 6393 bp from the 3'UTR end were selected. The last informative SNP with 0.349 as MAF was at the position 117,307,108 that corresponded to the last nucleotide of the CFTR insertion 4428insGA according to the website Clinical and Functional Translation of CFTR [8].

SNP primer sequences are listed in supplementary table 1.

### Patients undergoing PGT, ovarian stimulation, ICSI on fresh, and vitrified/warmed oocytes, embryo culture

Seventeen couples underwent 18 PGT-CF. Sixteen couples were both carriers, and 1 couple was comprised of a patient heterozygote compound (F508del;L206W, couple O in Table 3) and a carrier (R553X/N). At the time of PGT-CF, the female patients were aged between 30.0 and 39.3 years (mean age 32.1 years), with basal FSH on day 3 between 2.9 and 12.0 IU/l (mean 4.8 IU/l). All patients had a normal karyotype. Five patients underwent one cycle of ovarian stimulation to vitrify and accumulate oocytes and a second cycle of ovarian stimulation to use oocytes as fresh to increase the number of oocytes and consequently of embryos to diagnose [28]. In 12 cycles, PGT was performed on embryos produced from fresh oocytes only.

The protocols of ovarian stimulation, ICSI, oocyte and embryo vitrification and warming, embryo culture and biopsy, endometrial preparation, and embryo transfer were previously described and are briefly resumed here [29].

Ovarian stimulation was performed by the administration of recombinant FSH and LH (Gonal-F and Luveris; Merck-Serono, London, UK or Puregon, MSD, Franklin Lakes, USA) from cycle day 2–3 and luteal gonadotrophin-releasing hormone antagonist flexible schema (Cetrotide; Merck-Serono, London, UK or Orgalutran, MSD, Ireland). Vaginal ultrasound-guided aspiration of oocyte−cumulus complex (OPU) was performed 35 h after human chorionic gonadotrophin administration (HCG 10,000 IU, Gonasi; AMSA, Rome, Italy). Oocyte denudation was performed 2 h after OPU.

If oocytes had been accumulated by vitrification in dual stimulation protocol, ICSI was performed with the same partner’s sample on both fresh and accumulated vitrified/warmed oocytes. ICSI was performed 1 h after oocyte denudation on fresh oocytes and 1 h after warming and in vitro culture on vitrified/warmed oocytes.

In vitro culture was carried out in 25 μl of Continuous Single Culture Complete medium with human serum albumin (Irvine Scientific, Santa Ana, USA) under mineral oil and in automated incubators with 5% CO2, 5% O2 at 37 °C, fitted with time-lapse imaging acquisition (Embryoscope, Unisense, Aarhus, Denmark) until embryo biopsy.

### Embryo biopsy

Embryo biopsies were performed on day 5–6 on expanded or hatching blastocysts. As previously described [28, 29], the blastocyst was immobilized with a 120-um outer diameter holding micro-pipette in a 10-ul drop of HEPES buffered culture medium and under mineral oil. A few trophectoderm cells were removed. After the biopsy procedure, each embryo was washed in culture medium and incubated until embryo vitrification and before blastocyst re-expansion. The biopsied trophectoderm cells were washed in sterile phosphate buffered saline (PBS) solution and transferred into a 0.2 ml Eppendorf tube containing 4 ul of sterile PBS solution.

### Cell lysis, whole genome amplification and NGS protocol for PGT-CF and Data Analysis

The biopsied trophectoderm cells were submitted to alkaline lyses and whole genome amplification (WGA) according to the Repli-g Single Cell protocol (Qiagen, Hilden, Germany). From this part of the protocol, all products and devices were from Life Technologies-Thermo Fisher. The whole amplified DNA was quantified by the Qubit 2.0 fluorometer with the Qubit dsDNA HS assay kit.

A total of 5 ng of each multiple displacement amplification were submitted to multiplex PCRs with 101 primer pairs for the amplification of the total CFTR gene, according to the Ion Ampliseq CFTR Panel and 38 primer pairs for the amplification of the region with the informative SNPs. Multiplex PCRs were prepared using the Ion AmpliSeq Library Kit 2.0 according to the standard manufacturer’s workflow; DNA sequencing libraries were barcoded with the IonXpress Barcode Adapter Kit. After quantification, each library was normalized to 100 pM. All libraries were mixed to obtain a final concentration of 8 pM and clonally amplified with the Ion PGM™ Hi-Q™ View OT2 Kit on the Ion OneTouch 2 System. The enriched libraries were loaded on Chip 16 V2. DNA sequencing was performed with the Ion PGM™ Hi-Q™ View Sequencing Kit on the Ion Personal Genome Machine.

The alignments of tab-delimited text files (.BAM) containing sequences were performed as previously described in “Set-up for PGT-CF” paragraph. The specific hot spot .BED file was developed for the genomic call of each SNP, and the number of total readings was considered. It was possible to evade the phenomenon of allele drop out or contaminations by comparing the informative SNPs of the embryonic DNA of each biopsy with the DNA of the parents.

The diagnosis of wild-type, heterozygous, and double mutated was based on sequence results at the mutation point and the informative SNPs of the couples.

### PGT-A

The present protocol for PGT-A has been previously validated from single amniocytes with specific karyoptypes [28].

PGT-A was applied only to those embryos defined as transferable because they had previously been diagnosed as wild-type or a carrier for CF and from an aliquot of the previously whole genome amplified samples. The protocol of chromosomal analysis was previously described [28].

The previous products of WGA were processed using PGT-A according to the standard manufacturer’s protocol for IonXpress Plus Fragment Library Kit. After quantification of the previously amplified DNA, 100 ng of each library was prepared with the IonXpress Plus Fragment Library Kit and barcoded with the IonXpress Barcode Adapter kits. After quantification of the libraries, normalization to 100 pM and mixed to obtain a final concentration of 8 pM; the enriched libraries were loaded on Chip 16 V2. DNA sequencing was performed with the Ion PGM HiQ View Sequencing kit on Ion Personal Genome Machine. The updated Torrent Suite Software was used for base calling and mapping on human genome reference sequence hg19. For each chromosome, read coverage was corrected by guanine-cytosine calculation. Aneuploidy was tested comparing data with baseline values of multiple male samples. Throughout the process, a positive control with normal male DNA and a negative control from biopsy culture media were processed together with the samples to diagnose. The genetic analysis was processed using the dedicated workflow Reproseq Mosaicism Protocol with Ion Reporter Software and validated when the median absolute pairwise difference (MAPD) was inferior to 0.3. Chromosomal segments as short as 7 Mb could be detected. All products and devices used here were from Life Technologies-Thermo Fisher.

### Cell vitrification and warming and preparation for embryo transfer

Oocyte vitrification was started just after denudation and within 1 h of oocyte pick-up. The protocols of cell vitrification and warming were previously described [30, 31].

After warming, blastocyst re-expansion was verified before transfer. Single embryo transfers of diagnosed blastocysts were performed on a natural cycle at 7 days after the LH surge or on day 5 of progesterone administration after E2 priming in a hormonal replacement therapy cycle.

#### Statistical analysis

In case of a small sample size (*n* < 25), we can assume that the distribution of the population is normal. Consequently, the difference between the rates was checked by *z* test with *p* ˂ 0.05 due to the null hypothesis of no difference between the checked rates.

## RESULTS

### CF screening

According to the longitudinal screening for CF mutations of eastern infertile couples, it was calculated that 1 couple out of 105.2 of infertile Sicilian couples was at risk of having an affected child with CF (10/1052). Fifteen different mutations were found among the 17 Sicilian couples (infertile and fertile) and were tested at preimplantation stage.

### PGT-CF and PGT-A

All couples underwent PGT-CF combined with PGT-A on fresh (group I) or fresh and vitrified/warmed oocytes (group II).

### In vitro results

The results of ovarian stimulations, oocyte vitrification for accumulation, ICSI, and embryo culture for PGT-CF + PGT-A cycles on fresh and vitrified/warmed oocytes are presented in Part 1-A of Table 1. Seventeen patients underwent a total of 23 ovarian stimulations. A total of 262 metaphase II oocytes were produced. Thirty-seven oocytes were vitrified and 33 survived after warming (survival rate 89.2%). After ICSI, the fertilization rate was 83.5% (147/176) for group I; and respectively, 72.7% (24/33) and 67.3% (33/49) for the vitrified/warmed accumulated oocytes and the fresh oocytes from the same patients in group II (*p* > 0.05).

**Table 1:** In vitro results (Part 1-A), PGT-CF results (Part 1-B), PGT-A results (Part 1-C) on transferrable embryos after PGT-CF, and clinical outcomes (Part 1-D) for patients from group I (cycles from fresh oocytes only) and patients from group II (cycles from vitrified/warmed oocytes in addition with fresh oocytes)

	Group I	Group II		Total *p*
	Fresh oocytes (one ovarian stimulation)	Vitrified/warmed oocytes (1st ovarian stimulation)	Fresh oocytes (2nd ovarian stimulation)	
N. patients	12*	5		17
Mean patient age at PGT time	32.2	32.0		
In vitro results (Part 1-A)				
N. ovarian cycles	13	5	5	23
Metaphase II oocytes at OPU (mean per OPU)	176 (13.5)	37 (7.4)	49 (9.8)	262
Vitrified oocytes	-	37	-	37
Survived oocytes (survival rate)	-	33 (89.2%)	-	33
Micro-injected oocytes (ICSI)	176	33	49	258
Zygotes (fertilization rate)	147 (83.5%)	24 (72.7%)	33 (67.3%)	204 NS**
Expanded or hatching biopsied blastocysts (proportion on zygote)	82 (55.8%)	9 (37.5%)	20 (60.6%)	111 < 0.05**
Vitrified biopsied blastocysts (percentage from micro-injected MII oocyte)	81 (46.0%)	9 (27.3%)	19 (38.8%)	109 (42.2%) NS**
PGT-CF (Part 1-B)				
PGT-CF (with or without PGT-A)	13	5		18
Started analyses (biopsied blastocysts to analyse)	81	9	19	109
Conclusive genetic analysis (percentage)	75 (92.6%)	8 (88.9%)	18 (94.7%)	101 (92.7%) NS**
N. wild-type	16	2	3	21 (20.8%)
N. heterozygous	33	1	9	43 (42.6%)
N. affected	26	5	6	37 (36.6%)
Number of transferrable embryos (percentage on biopsied and vitrified embryos)	49 (60.5%)	3 (33.3%)	12 (63.2%)	64 (58.7%) NS**
PGT-A (Part 1-C)				
Started chromosomal analyses on transferrable embryos after PGT-CF	49	3	12	64
Conclusive genetic analysis (percentage)	48 (98.0%)	3 (100%)	10 (83.3%)	61 (95.3%)
N. euploid blastocysts (euploidy rate)	29 (60.4%)	2 (66.6%)	7 (70.0%)	38 (62.3%)
N. aneuploid blastocysts (aneuploidy rate)	19 (39.6%)	1 (33.3%)	3 (30.0%)	23 (37.7%)
Number of transferrable embryos (Percentage on biopsied and vitrified embryos)	29 (35.8%)	2 (22.2%)	7 (36.8%)	38 (34.9%)
Clinical outcomes (Part 1-D)				
N. embryo transfers	13	1	2	16
N. transferred embryos	13	1	2	16
N. implanted embryos (implantation rate)	10 (76.9%)	1 (100%)	2 (100%)	13 (81.3%)
N. arrested pregnancies	1 (10.0%)	0	0	1 (7.7%)

^*^One couple underwent two PGT cycles.

^**^Comparing vitrified/warmed and fresh oocytes from the same patients

After embryo culture, until day five, 55.8% (82/147) of the zygotes reached the expanded or hatching blastocyst stage available to be biopsied in group I. Respectively, 37.5% (9/24) of the zygotes produced from vitrified/warmed oocytes and 60.6% (20/33) of the zygotes produced from fresh oocytes reached the expanded or hatching blastocyst stage available to be biopsied in group II (*p* < 0.05) (see Part 1-A of Table 1).

*NS* statistically non-significant

### Genetic results

The results of PGT-CF and PGT-A for groups I and II are presented in respectively Parts 1-B and 1-C of Table 1.

In a first time, PGT-CF was performed on all samples from all vitrified blastocysts. The genetic analysis was completed and conclusive in 92.7% (101/109) of the started analyses (respectively, 75/81 in group I, 8/9 in blastocysts from vitrified/warmed oocytes 18/19 in blastocysts from fresh oocytes in group II *p* > 0.05). It resulted that 20.8% (21/101) of them were diagnosed as wild-type, 42.6% (43/101) as heterozygous, and 36.6% (37/101) as double mutated (Part 1-B of Table 1).

Analyzing the 8/109 analyses that failed, it resulted that 4 were due to only one allele amplification (allele drop out) and 4 to the failure of both allele amplifications. The full amplification failure was defined when all the general parameters did not meet the following: uniformity of the coverage, percent reads on target, average base coverage depth, and number of cases of mapped reads superior to 10,000. Out of the 218 allele amplifications, the allele no-amplification rate was 5.5% (12/218). The analysis of CFTR amplification failures is described in Table 2.

**Table 2:** Analysis of CFTR amplification failure.

	Total	Alleles
Started analysis (embryos to analyse)	109	218
Conclusive (completed) analysis	101 (92.7%)	
Uncompleted analysis	8 (7.3%)	
Samples with no amplification	4	8
Samples with ADO	4	4
Samples with ADO and transferrable	1	
Samples with ADO and not transferrable	3	
Allele no-amplification rate		12/218=> 5.5%

ADO (allele drop-out).

PGT-A was performed on embryos resulting as transferrable after PGT-CF (wild-type and heterozygous). Consequently, PGT-A was performed on 64 blastocysts (49 in group I and 15 in group II). The genetic analysis was completed in 95.3% (61/64) of the started analyses. Altogether, 62.3% (38/61) of the blastocysts resulted as euploid and transferrable after PGT-A (respectively 29/48 in group I and 9/13 in group II), and 37.7% (23/61) were aneuploid (respectively 19/48 in group I and 4/13 in group II) (Part 1-C of Table 1).

In conclusion, after PGT-CF and PGT-A, 34.9% (38/109) of the biopsied and vitrified embryos were transferable (respectively 29/81 in group I and 9/28 in group II; *p* > 0.05).

The SNPs defined as informative during set-up in the pre-PGT-CF phase were amplified together with the CFTR gene from the DNA-produced WGA of biopsied trophectoderm cells. Within the 17 couples undergoing PGT-CF, 5 SNPs were informative in all cases (100% of informative frequency), 18 SNPs with a frequency between 76.5 and 96.1% (13/17 to 16/17 cases), 18 SNPs with a frequency between 52.9% and 5.9% (9/17 to 1/17), and 2 SNPs were not informative. The number of informative SNPs per couple was between 14 and 28, with a mean of 24.1.

The cartography of CFTR gene mutations and informative SNPs for each couple undergoing PGT-CF is described in Table 3.

**Table 3:**
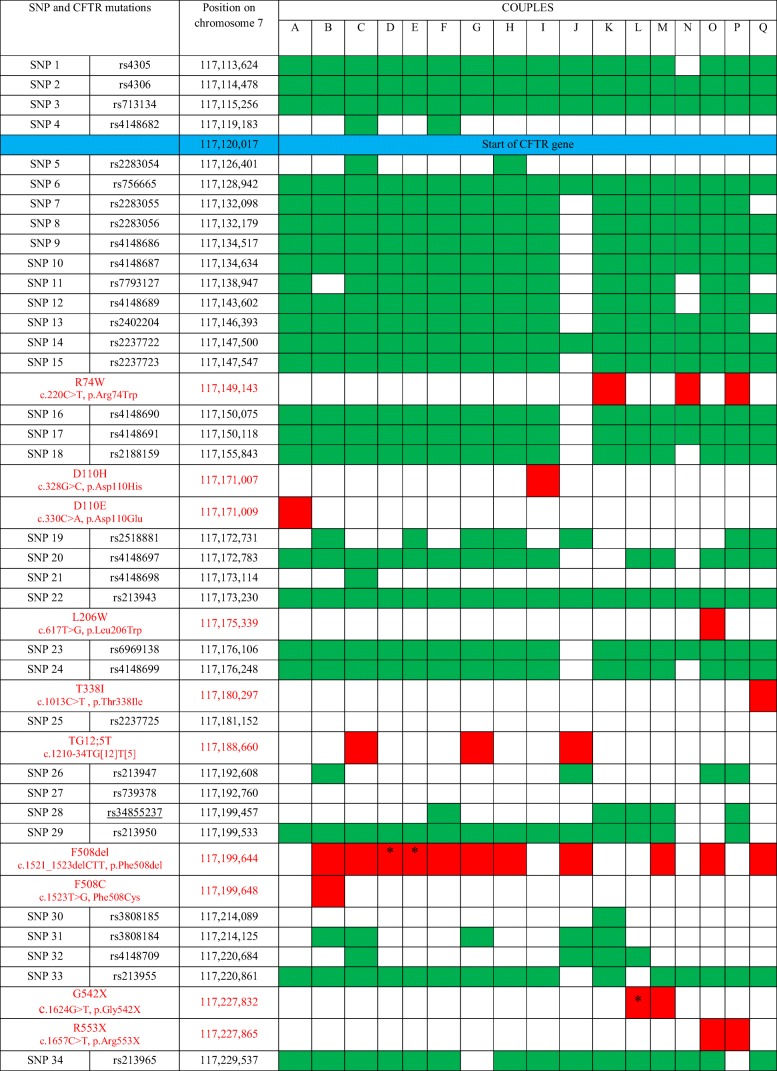

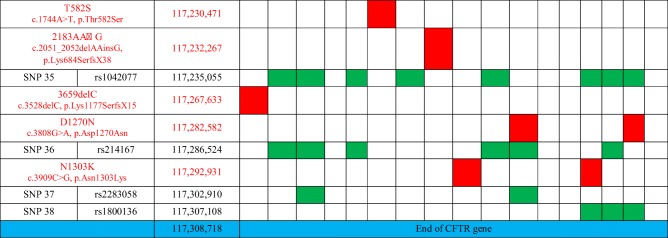
Cartography of CFTR gene mutations and informative SNPs for each Sicilian couple candidate to PGT-CF

In the supplementary figure 1 (1A and 1B), the allele segregations with CFTR mutations and informative SNPs are illustrated for families A and I. Both wild-type and mutated alleles are followed through mutation detection and linkage analysis.

### Clinical outcomes

A total of 16 euploid and wild-type or heterozygous for CF embryos produced from fresh or vitrified/warmed oocytes were warmed and transferred singularly. Thirteen embryos were implanted, giving 13 clinical pregnancies. One pregnancy was arrested at the 7th week. All the PGTs were confirmed during prenatal diagnosis by chorionic villus sampling or amniocentesis. Twelve healthy babies were born.

The clinical outcomes are described in Part 1-D of Table 1.

## DISCUSSION

### CF Screening

Among the 1052 infertile Sicilian couples tested in our laboratory for longitudinal screening of all CF mutations through NGS, 10 couples (1/105.2) were at risk of the transmission of CF. Twenty-two different CFTR mutations were observed (data not shown). Fifteen mutations were tested at preimplantation stage on embryos from fertile and infertile couples from eastern Sicily.

### Universal strategy for PGT-CF

CF has been the first disease tested by PGT [19] and remains among the widespread diseases tested at preimplantation stage.

At the beginning of clinical application of PGT-CF, clinical analyses were based on single-target amplification [32], then multiplex PCR [33] and mini-sequencing strategy [34] were developed. At this point, it became possible to amplify different target genes together with specific polymorphisms allowing DNA contamination detection and following gene segregation directly (mutation point) and indirectly (linkage analysis). With the introduction of WGA in NGS by multiple displacement amplifications [35], gene testing of a long sequence such as the CFTR gene compound of 27 exons together with aneuploidy, genetic, and chromosomal testing from few embryonic cells became possible. Consequently, it appeared possible to set-up molecular protocols in order to diagnose all CF mutations in a single run and making at the same time an intrinsic validation of the test by the cartography of specific and informative SNPs. This is the strategy that we applied here: 4 SNPs upstream of the CFTR gene and 34 SNPs internal to the gene were integrated to gene analysis in order to trace the wild-type and mutated alleles. In the set-up of universal strategy for the diagnosis of CF mutations, NGS is superior to the previously gold-standard methods such as mini-sequencing because a high number of informative polymorphisms can be tested in a single run, and both PGT-A and PGT-CF can be performed in the same machine at the meantime.

In the present work, the CFTR diagnosis was conclusive at 92.7% from the first biopsy. In 8 samples, there was no amplification of chromosome 7. Based on trophectoderm cell analysis, testing directly the presence/absence of the mutated genes and indirectly by SNP linkage, 63.4% of the embryos resulted as being transferrable (wild-type or carrier). The number of informative SNPs was between 14 and 28, with a mean value of 24.1 per couple. The maximum distance between CFTR mutation locus and the farthest informative SNP was 63 Kb (couple I, SNP 34). The number of informative SNPs is directly dependent on the allele diversity between each member of the couple, and it is not predictable a priori. This is the reason why PGT-CF must be preceded by a set-up phase, researching for informative SNPs from the couple’s blood and direct relatives of both members. In the latest best practice guidelines for PGT-CF, it was recommended to include at least 4 informative microsatellite markers (four per allele) within 1 Mb of distance to the gene when WGA was performed by multiple displacement amplification as it was here [24]. The present protocol surpassed the minimal requested conditions. The CFTR panel applied here (Ion Ampliseq CFTR panel) can detect all mutations except the large deletions with an allele frequency inferior or equal to 0.00044 as so defined as the rarest ones [8]. The only detectable large deletions are CFTRdele2,3 and CFTRdele22,23. Nevertheless, applying the present strategy based on tested SNPs covering all the CFTR gene and evenly distributed from the upstream position to the last nucleotide (position 117,307,108) of the last CFTR mutation (4428insGA), it is possible to follow allele segregation directly (point mutation and linkage analysis) or indirectly (linkage analysis only). Consequently, all CF mutations can be diagnosed at preimplantation stage with the present strategy.

Based on mutation and SNP amplification, it was calculated that allele amplification was 94.5%. Two of the tested SNPs were not informative even if MAF was between 0.286 and 0.369. Our amplification failure was 5.5%. Kubikova et al. [36] applied the same molecular strategy with an allele amplification efficacy of 9.5 % for β-globin mutations diagnosis (42 alleles 2 ADO, an amplification failure). When requested, PGT-A was performed with an efficacy of 91.7% and the 55.6% blastocysts resulted as euploid.

As previously published [28], the strategy of oocyte accumulation was applied here to increase the number of blastocysts to biopsy in group II. Fertilization rates were statistically inferior for vitrified/warmed oocytes compared with fresh oocytes from the same patients. Consequently, the number of expanded/hatching biopsied blastocysts and the number of vitrified biopsied and tested embryos were inferior (even if not statistically significant) from vitrified/warmed oocytes compared with fresh oocytes.

After PGT-A on transferrable embryos according to PGT-CF, the ratio of transferrable embryos decreased from 58.7% of the biopsied and vitrified embryos according to PGT-CF to 34.9% after PGT-A. After single embryo transfer, implantation rate was 81.3 %.

## Conclusion

CF is the most widespread autosomal recessive disease present in the Caucasian population with a very large variability as 383 mutations have been lastly identified, and many genomic variations have uncertain clinical consequences. In our tested Sicilian infertile population, 1/105.2 couple was at risk of CF transmission.

We developed and successfully applied a universal strategy based on NGS for PGT of all CF mutations based on SNP segregation along the entire CFTR gene to detect all the wild-type and carrier embryos to transfer. The present strategy based on sequencing on the entire gene together with informative SNPs outside and inside the gene as applied here for the first time makes PGT applicable for any multi-allele genetic disease.

## Electronic supplementary material

ESM 1 (DOCX 157 kb)

ESM 2 (DOCX 17.5 kb)
