# Supplementary material for: Universal strategy for preimplantation genetic testing for cystic fibrosis based on next generation sequencing
Source: J Assist Reprod Genet. 2019 Dec 11;37(1):213–22. doi: 10.1007/s10815-019-01635-2 (PMC7000499; doi:10.1007/s10815-019-01635-2)
Supplement: Supplementary file 1 — (DOCX 157 kb) [file 10815_2019_1635_MOESM1_ESM.docx]

**Supplementary Figure 1A** : Allele segregation of CFTR gene according to mutation detection and informative SNPs in family A.

**Supplementary Figure 1B** : Allele segregation of CFTR gene according to mutation detection and informative SNPs in family I.

Yellow colour: informative SNP for mutated and wild-type alleles for the one parent only. Blu colour: informative SNP for mutated and wild-type alleles for both parent. Red colour: mutation.
