# Supplementary material for: Universal strategy for preimplantation genetic testing for cystic fibrosis based on next generation sequencing
Source: J Assist Reprod Genet. 2019 Dec 11;37(1):213–22. doi: 10.1007/s10815-019-01635-2 (PMC7000499; doi:10.1007/s10815-019-01635-2)
Supplement: Supplementary file 2 — (DOCX 17.5 kb) [file 10815_2019_1635_MOESM2_ESM.docx]

**Supplementary Table 1**: SNP: name, position on chromosome 7, alleles, minor allele frequency, primer sequences, amplicon size, informative frequency in the PGT-CF candidate couples.

| SNP | | Position | Alleles | MAF | Forward primer | Reverse primer | Amplicon size | Informative frequency  n (%) |
| --- | --- | --- | --- | --- | --- | --- | --- | --- |
| 1 | rs4305 | 117,113,624 | A/C | 0.495 | TTTTTCTTACAAAGACGGTACCATACTAAA | GCTGTTGAGAGGCAACACTGA | 217 | 16 (94.1) |
| 2 | rs4306 | 117,114,478 | C/T | 0.495 | TGTCTCTCTTATTATTTCAAATGCCCTGTT | GGTGATATCATGCTAAAGGCCAGAT | 203 | 17 (100) |
| 3 | rs713134 | 117,115,256 | C/T | 0.492 | TAGCTTTTAGAGAGTTCAGTTTTCTGTAGC | TCCTAGGTTGTGACAGATAGATCTGG | 216 | 17 (100) |
| 4 | rs4148682 | 117,119,183 | T/G | 0.302 | CCTTCGCTTCAAAGACTGCTTG | GCACAGAAATCTTAGGACACGTACT | 228 | 2 (11.8) |
| 5 | rs2283054 | 117,126,401 | G/A | 0.304 | TATGCCTGGATATAGCCAGAAAACTCT | CCTTCCTTGACCAGCCTTAGAC | 226 | 2 (11.8) |
| 6 | rs756665 | 117,128,942 | A/C | 0.498 | AAAGAAACAGTTAACCTAGTGCTCTAAAGT | GTGGGATGCAACCAATGCAAAT | 211 | 17 (100) |
| 7 | rs2283055 | 117,132,098 | T/A | 0.394 | CCTCATGTATAGTGGTCCATAAATAGCATG | TCATCCTAAAACTTCCAGCCAACATT | 219 | 15 (88.3) |
| 8 | rs2283056 | 117,132,179 | A/G | 0.400 | CCTCATGTATAGTGGTCCATAAATAGCATG | TCATCCTAAAACTTCCAGCCAACATT | 219 | 16 (94.1) |
| 9 | rs4148686 | 117,134,517 | C/G | 0.415 | CGCTTTGCCTGTAGTGGTTCAT | TTCAGATACCATCACTAGGACCCAA | 217 | 16 (94.1) |
| 10 | rs4148687 | 117,134,634 | G/A | 0.401 | CGCTTTGCCTGTAGTGGTTCAT | TTCAGATACCATCACTAGGACCCAA | 217 | 16 (94.1) |
| 11 | rs7793127 | 117,138,947 | A/G | 0.415 | TCTTTCCTCTGCCTGTCTTCAAC | GGAAATTCAAACCACTAATTGCATGACA | 223 | 13 (76.5) |
| 12 | rs4148689 | 117,143,602 | C/G | 0.415 | CTCTCGTGCTCTCGAATTTTATTTAGAAAT | CCTCACTCCATAGCAGCTACACT | 222 | 15 (88.3) |
| 13 | rs2402204 | 117,146,393 | T/C | 0.415 | ACATAATCCATTGCAGGTATATTGACAAGT | ATGGCGAAACTCCACATCTACTAAAAT | 218 | 15 (88.3) |
| 14 | rs2237722 | 117,147,500 | C/G | 0.495 | CTCTCCTGGTCTCTCTACTTCACTTT | TACTCAGAGTAAGAAGCTAAATATGGAGCA | 219 | 17 (100) |
| 15 | rs2237723 | 117,147,547 | C/T | 0.395 | CTCTCCTGGTCTCTCTACTTCACTTT | TACTCAGAGTAAGAAGCTAAATATGGAGCA | 219 | 16 (94.1) |
| 16 | rs4148690 | 117,150,075 | C/A | 0.402 | AATGCCATTTGTGTTACATTGCTTGA | CCCAAATGCCCAAATGGTTTCA | 167 | 16 (94.1) |
| 17 | rs4148691 | 117,150,118 | G/C | 0.417 | AATGCCATTTGTGTTACATTGCTTGA | CCCAAATGCCCAAATGGTTTCA | 167 | 16 (94.1) |
| 18 | rs2188159 | 117,155,843 | A/G | 0.416 | CTGCACCATTGGTCTATATGTCTGTTAT | ACATGGCTATACTGCCCAAAGTAATTTATAAT | 198 | 15 (88.3) |
| 19 | rs2518881 | 117,172,731 | A/G | 0.370 | ACTGATATTTAGTGGTCAGACTGTAATGC | TGATGATGCCATTGTAGGCCAA | 222 | 7 (41.2) |
| 20 | rs4148697 | 117,172,783 | C/T | 0.416 | ACTGATATTTAGTGGTCAGACTGTAATGC | TGATGATGCCATTGTAGGCCAA | 222 | 14 (82.4) |
| 21 | rs4148698 | 117,173,114 | T/C | 0.286 | GGACCTTTCCTACAATGTTCCAAAGTT | GGGAAATCCACTGAGCTAAATTGC | 224 | 1 (5.9) |
| 22 | rs213943 | 117,173,230 | T/C | 0.497 | GGACCTTTCCTACAATGTTCCAAAGTT | GGGAAATCCACTGAGCTAAATTGC | 216 | 17 (100) |
| 23 | rs6969138 | 117,176,106 | A/G | 0.402 | TGTGTAGAGTTTATTAGCTTTTACTACTCTGGT | AAAAAGCAAAGACTCAGTGAGGT | 216 | 16 (94.1) |
| 24 | rs4148699 | 117,176,248 | A/G | 0.402 | TGTGTAGAGTTTATTAGCTTTTACTACTCTGGT | AAAAAGCAAAGACTCAGTGAGGT | 216 | 15 (88.3) |
| 25 | rs2237725 | 117,181,152 | A/G | 0.286 | GCAGCCTCTTTTGAAGAATTGGAATAAC | TCTCTCTATTTCTCTCTCAATGGGCTAA | 219 | 0 (0) |
| 26 | rs213947 | 117,192,608 | A/G | 0.238 | ATCTGTTACATGACCTTCCTTTCTTTTGA | ACCTTGGTTGGATGAGGGAATG | 116 | 4 (23.5) |
| 27 | rs739378 | 117,192,760 | A/C | 0.289 | TTTACTGTCTCTCATCTGTCCATTTTCC | GGAGAGACAGAAATATTCAGCAGTCT | 221 | 0 (0) |
| 28 | [rs34855237](https://www.ncbi.nlm.nih.gov/projects/SNP/snp_ref.cgi?rs=34855237) | 117,199,457 | G/A | 0.336 | GTATCTTTTGTGCATAGCAGAGTACCT | TTCTTCCACTGTGCTTAATTTTACCCT | 196 | 5 (29.4) |
| 29 | rs213950 | 117,199,533 | G/A | 0.497 | GTATCTTTTGTGCATAGCAGAGTACCT | TTCTTCCACTGTGCTTAATTTTACCCT | 129 | 14 (82.4) |
| 30 | rs3808185 | 117,214,089 | T/C | 0.369 | TTCCCACAACAATTTTTGTGGATAACT | TTGCTGATTACTAAGAGCGCTCAA | 224 | 1 (5.9) |
| 31 | rs3808184 | 117,214,125 | T/C | 0.460 | TTCCCACAACAATTTTTGTGGATAACT | TTGCTGATTACTAAGAGCGCTCAA | 224 | 5 (29.4) |
| 32 | rs4148709 | 117,220,684 | G/A | 0.286 | AACGCATACTTTTGACCTAGCCAT | TATGAAACCTGCCTTCAAGCACT | 228 | 4 (23.5) |
| 33 | rs213955 | 117,220,861 | A/G | 0.487 | TGAACTACAAAAGCTTACACTTTCAGTCT | CCCACACTTGTTGCTAATTGAATAACA | 176 | 15 (88.2) |
| 34 | rs213965 | 117,229,537 | T/A | 0.498 | TGGCTTTAAAAATTTCTTAATTGTGTGCTGA | CAGTAATAAAGATGAAGACACAGTTCCCA | 143 | 15 (88.2) |
| 35 | rs1042077 | 117,235,055 | T/G | 0.493 | TGACTACATGGAACACATACCTTCGATA | TGTATACATCCCCAAACTATCTTAATTTAACTT | 140 | 8 (47.1) |
| 36 | rs214167 | 117,286,524 | G/A | 0.493 | CACAAAGAAAGTGATAATTAAGGCGGTT | AGCCTCTTCCTCACTCAAAAAGG | 224 | 6 (35.3) |
| 37 | rs2283058 | 117,302,910 | A/C | 0.302 | TGATGATGACCCAGACTGGGAT | GCTTGAGAGAAACCACACACTCT | 230 | 2 (11.8) |
| 38 | rs11800136 | 117,307,108 | G/A | 0,349 | TGCTAAGGTAGGTAGGTCTTTGAC | AGCTCCAATTCCATGAGC | 202 | 3 (17.5) |
